# Prognostic Nomogram for Early Gastric Cancer After Surgery to Assist Decision-Making for Treatment With Adjuvant Chemotherapy

**DOI:** 10.3389/fphar.2022.845313

**Published:** 2022-04-08

**Authors:** Chao Zhang, Shutao Zhao, Xudong Wang

**Affiliations:** Department of Gastrointestinal Nutrition and Hernia Surgery, The Second Hospital of Jilin University, Changchun, China

**Keywords:** early gastric cancer, nomogram, overall survival, postoperative adjuvant chemotherapy, prognosis

## Abstract

**Background:** Most patients with early gastric cancer (EGC) can achieve a better 5-year survival rate after endoscopic resection or surgery. However, indications for adjuvant chemotherapy (ACT) after surgery have not yet been determined.

**Methods:** A total of 4,108 patients with EGC diagnosed in 2004–2016 were retrospectively analyzed using the Surveillance, Epidemiology, and End Results (SEER) database. Of these, 3,521 patients received postoperative ACT and 587 patients did not. Propensity score matching was used to balance the two groups’ confounding factors. Kaplan-Meier method was utilized to perform survival analysis. Log-rank test was used to compare the differences between survival curves. Cox proportional-hazards regression model was used to screen independent risk factors and build a nomogram for the non-ACT group. The X-tile software was employed to artificially divide all patients into low-, moderate-, and high-risk groups according to the overall survival score prediction based on the nomogram. A total of 493 patients with EGC diagnosed between 2010 and 2014 in our hospital were included for external validation.

**Results:** Multivariate analysis found that age, sex, race, marital status, primary site, surgical extent, and metastatic lymph node ratio in the non-ACT group were independent prognostic factors for EGC and were included in the construction of the nomogram. The model C-index was 0.730 (95% confidence interval: 0.677–0.783). The patients were divided into three different risk groups based on the nomogram prediction score. Patients in the low-risk group did not benefit from ACT, while patients in the moderate- and high-risk groups did. External validation also demonstrated that moderate- and high-risk patients benefited from ACT.

**Conclusion:** The study nomogram can effectively evaluate postoperative prognosis of patients with EGC. Postoperative ACT is therefore recommended for moderate- and high-risk patients, but not for low-risk patients.

## Background

Early gastric cancer (EGC) is defined as a tumor confined to the mucosa or submucosa, regardless of lymph node metastasis. This concept was first proposed by the Japanese Gastric Cancer Research Association in 1962 and has been used up until now (Katai and Sano, 2005). For EGC patients who do not have lymph node metastasis and meet the indications for endoscopic mucosal resection and endoscopic submucosal dissection, minimally invasive endoscopic treatment can completely remove the lesions (Lian et al., 2012; El-Sedfy et al., 2014; Ono et al., 2016; Suzuki et al., 2016; Tanoue et al., 2019). Although endoscopic resection is the preferred option for EGC patients in accordance with indications, the impossibility of regional lymph node clearance is a serious limitation. Approximately 8.9–15.8% of EGC cases will have regional lymph node metastasis, which may lead to tumor recurrence and the need for invasive radical gastrectomy (Roviello et al., 2006; Pessorrusso et al., 2018). Radical surgical treatment can achieve sufficient tumor clearance and lymph node dissection range and its recurrence rate is low, with a 5-year survival rate of >90% (Zeng et al., 2012). The risk of postoperative recurrence in EGC with lymph node metastasis is high, and it is still unclear whether postoperative adjuvant chemotherapy (ACT) can benefit patients with a high risk of such recurrence and poor prognosis.

Clinical research on gastric cancer ACT is mainly focused on advanced gastric cancer, while research on EGC ACT alone is less prevalent (Cai et al., 2018; Yoshida et al., 2019). Although the NCCN guidelines recommend that patients at T stage accompanied by N + should receive ACT after surgery (Ajani et al., 2013), the Japanese guidelines for the treatment of gastric cancer recommend postoperative observation of EGC patients (Japanese gastric cancer, 2017). There are few clinical trials on ACT indications, which makes it more difficult to make postoperative treatment decisions for EGC patients.

A nomogram is a scoring tool used to quantify the likelihood of clinical events in combination with a variety of factors, which has been widely accepted internationally in recent years. Studies have shown that a nomogram is more accurate in assessing clinical events than a traditional prediction model (Balachandran et al., 2015; Hoban et al., 2018). However, a nomogram for predicting overall survival (OS) for EGC is still not established. Therefore, this study aimed to establish a nomogram that can evaluate the prognosis EGC patients based on a variety of clinicopathological factors in the Surveillance, Epidemiology, and End Results (SEER) database and to solve the problem of patient selection for EGC ACT.

## Methods

### Patient Cohort

**FIGURE 1:**
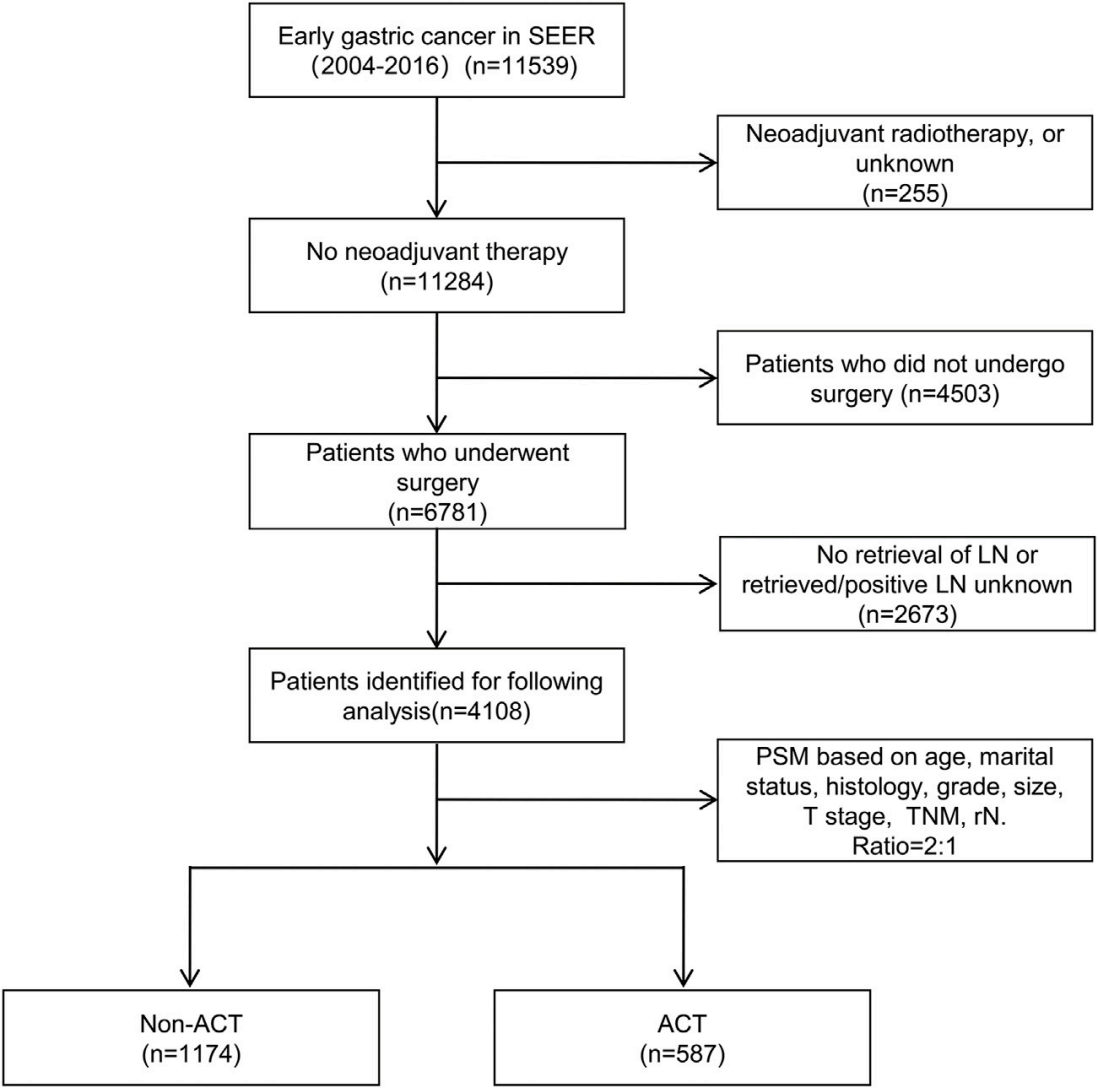
Flowchart of the selection process of included patients.

SEER*Stat (version 8.3.6) software was used to search for 4,108 patients diagnosed with EGC (T1N0-3M0) after surgery performed in 2004–2016. First, positive histology was selected to ensure a correct diagnosis. Active follow-up and complete survival time data were selected, and the source of cases excluded those obtained from autopsy and death certificate only. The remaining inclusion and exclusion criteria are shown in Figure 1. The patients were divided into two groups according to whether they received postoperative ACT: the non-ACT (3,521) and ACT (587) groups. The variables included age, sex, race, marital status, primary site, history, grade, size, surgical extent, T stage, metastatic lymph node ratio (rN), ACT information, and survival information. Patients with unknown surgical status or endoscopic resection, and missing information on the above variables were excluded.

External validation data were collected from patients with EGC in our hospital between 2010 and 2014. According to the above criteria, 493 patients were included in the final analysis.

### Statistical Analysis

The chi-square test was used to compare the baseline data characteristics in the ACT and non-ACT groups. In order to reduce the confounding bias of the included cases, meaningful clinical pathological factors of the chi-square test were included in propensity score nearest-neighbor matching (PSM) analysis, where the non-ACT and ACT groups were matched 2:1 (Austin, 2011). Then, Kaplan-Meier method and log-rank test were used to obtain survival information.

The following steps were taken to establish a predictive model (Katai and Sano, 2005): Use univariate analysis to analyze the correlation between OS and variables (Lian et al., 2012); incorporate variables with statistical differences in univariate analysis (*p* < 0.05) into the Cox proportional-hazards model (Ono et al., 2016); establish a nomogram based on the Cox proportional-hazards model; and (Suzuki et al., 2016) test the effectiveness of the prediction model. The concordance index (C-index) is used to measure the degree of discrimination (Hanley and McNeil, 1983). (Tanoue et al., 2019) A calibration curve was obtained by resampling the data 1,000 times. The calibration curve visually demonstrated the consensus degree between the predicted survival rate and the actual survival rate while avoiding model overfitting (El-Sedfy et al., 2014). Decision curve analysis (DCA) was used to evaluate the net clinical benefit (Pessorrusso et al., 2018). X-tile software was used to artificially divide the cases into three groups of low-, moderate-, and high-risk according to the predicted OS score based on the nomogram (Camp et al., 2004). All statistical analyses in this study were conducted using SPSS 24.0 and R software (version 3.6.3), and *p* < 0.05 was considered statistically significant.

## Results

### Patient Demographics

According to the inclusion and exclusion criteria (Figure 1), a total of 4,108 EGC patients were evaluated before PSM analysis, including 3,521 in the non-ACT and 587 in ACT groups. The median patient survival time was 51 months (0–155), and the number of deaths was 1,508 (36.7%). The clinicopathological data showed that ACT was significantly correlated with age, marital status, histology, grade, size, T stage, and rN (*p* < 0.05). When these ACT-related variables were included in PSM analysis, the final matched population was 1,761, including 1,174 in the non-ACT and 587 in the ACT groups (Table 1). The median patient survival time was 50 months (0–155), and the number of deaths was 618 (35.1%).

**TABLE 1:** Characteristics of patients.

Variable	Unmatched cohort			*p* Value	Matched cohort			*p* Value
	Total [*n* (%)]	Non-ACT [*n* (%)]	ACT [*n* (%)]		Total [*n* (%)]	Non-ACT [*n* (%)]	ACT [n (%)]	
Age	—	—	—	<0.001	—	—	—	<0.001
<65	1,383 (33.6)	1,098 (31.2)	285 (48.6)	—	999 (56.7)	714 (60.8)	285 (48.6)	—
≥65	2,725 (66.4)	2,423 (68.8)	302 (51.4)	—	762 (43.3)	460 (39.2)	302 (51.4)	—
Sex				0.882				0.580
Male	2,466 (60.0)	2,112 (59.9)	354 (60.3)	—	1,078 (61.2)	724 (61.7)	354 (60.3)	—
Female	1,642 (40.0)	1,409 (50.1)	233 (39.7)	—	683 (38.8)	450 (38.3)	233 (39.7)	—
Race	—	—	—	0.648	—	—	—	0.389
White	2,575 (62.7)	2,209 (62.7)	366 (62.4)	—	1,121 (63.7)	755 (64.3)	366 (62.4)	—
Black	515 (12.5)	435 (12.4)	80 (13.6)	—	236 (13.4)	156 (13.3)	80 (13.6)	—
API	992 (24.1)	853 (24.2)	139 (23.7)	—	391 (22.2)	252 (21.5)	139 (23.7)	—
Other	26 (0.7)	24 (0.7)	2 (0.3)	—	13 (0.7)	11 (0.9)	2 (0.3)	—
Marital status	—	—	—	0.001	—	—	—	0.084
Married	2,899 (70.6)	2,450 (69.6)	449 (76.5)	—	1,347 (76.5)	898 (76.5)	449 (76.5)	—
Unmarried	470 (11.4)	409 (11.6)	61 (10.4)	—	215 (12.2)	154 (13.1)	61 (10.4)	—
Unknown	739 (18.0)	662 (18.8)	77 (13.1)	—	199 (11.3)	122 (10.4)	77 (13.1)	—
Primary site				0.645				0.646
Cardia	977 (23.8)	839 (23.8)	138 (23.5)	—	442 (25.1)	304 (25.9)	138 (23.5)	—
Fundus	120 (2.9)	102 (2.9)	18 (3.1)	—	54 (3.1)	36 (3.1)	18 (3.1)	—
Body	464 (11.3)	391 (11.1)	73 (12.4)	—	216 (12.3)	143 (12.2)	73 (12.4)	—
antrum	1,300 (31.6)	1,126 (32.0)	174 (29.6)	—	518 (29.4)	344 (29.3)	174 (29.6)	—
Pylorus	125 (3.0)	105 (3.0)	20 (3.4)	—	48 (2.7)	28 (2.4)	20 (3.4)	—
Lesser curve	459 (11.2)	399 (11.3)	60 (10.2)	—	187 (10.6)	127 (10.8)	60 (10.2)	—
Greater curve	222 (5.4)	182 (5.2)	40 (6.8)	—	98 (5.6)	58 (4.9)	40 (6.8)	—
Overlapping/not otherwise specified	441 (10.8)	377 (10.7)	64 (11.0)	—	198 (11.2)	134 (11.4)	64 (11.0)	—
Histology	—	—	—	0.018	—	—	—	0.908
Adenocarcinoma	3,169 (77.1)	2,741 (77.8)	428 (72.9)	—	1,294 (73.5)	866 (73.8)	428 (72.9)	—
Signet ring cell carcinoma	685 (16.7)	574 (16.3)	111 (18.9)	—	323 (18.3)	212 (18.1)	111 (18.9)	—
Other	254 (6.2)	206 (5.9)	48 (8.2)	—	144 (8.2)	96 (8.1)	48 (8.2)	—
Grade	—	—	—	<0.001				<0.001
Well/Moderately	2036 (49.6)	1828 (51.9)	208 (35.4)	—	695 (39.5)	487 (41.5)	208 (35.4)	—
Poorly/undifferentiated	1878 (45.7)	1,514 (43.0)	364 (62.0)	—	961 (54.6)	597 (50.9)	364 (62.0)	—
Unknown	194 (4.7)	179 (5.1)	15 (2.6)	—	105 (5.9)	90 (7.6)	15 (2.6)	—
Size (cm)	—	—	—	<0.001	—	—	—	<0.001
≤2	2,281 (55.5)	2054 (58.3)	227 (38.7)	—	798 (45.3)	571 (48.6)	227 (38.3)	—
≤3	829 (20.2)	693 (19.7)	136 (23.2)	—	405 (23.0)	269 (22.9)	136 (23.2)	—
≤5	703 (17.1)	556 (15.8)	147 (25.0)	—	382 (21.7)	235 (20.0)	147 (25.0)	—
>5	295 (7.2)	218 (6.2)	77 (13.1)	—	176 (10.0)	99 (8.5)	77 (13.1)	—
Surgery	—	—	—	0.424	—	—	—	0.688
Partial	3,317 (80.7)	2,854 (81.1)	463 (78.9)	—	1,403 (79.7)	940 (80.1)	463 (78.9)	—
Near total/total	617 (15.0)	522 (14.8)	95 (16.2)	—	281 (16.0)	186 (15.8)	95 (16.2)	—
Unknown	174 (4.3)	145 (4.1)	29 (4.9)	—	77 (4.3)	48 (4.1)	29 (4.9)	—
T stage				<0.001				0.078
T1a	1,483 (36.1)	1,371 (38.9)	112 (19.1)	—	333 (18.9)	221 (18.8)	112 (19.1)	—
T1b	2,496 (60.8)	2063 (58.6)	433 (73.8)	—	1,332 (75.6)	899 (76.6)	433 (73.8)	—
T1 NOS	129 (3.1)	87 (2.5)	42 (7.1)	—	96 (5.5)	54 (4.6)	42 (7.1)	—
TNM stage	—	—	—	<0.001	—	—	—	<0.001
I	4,018 (97.8)	3,488 (99.1)	530 (90.3)	—	1,671 (94.9)	1,141 (97.2)	530 (90.3)	—
II	74 (1.8)	25 (0.7)	49 (8.3)	—	74 (4.2)	25 (2.1)	49 (8.3)	—
III	16 (0.4)	8 (0.2)	8 (1.4)	—	16 (0.9)	8 (0.7)	8 (1.4)	—
rN	—	—		<0.001	—	—	—	<0.001
0	3,301 (80.4)	3,139 (89.2)	162 (27.6)	—	954 (54.2)	792 (67.5)	162 (27.6)	—
0.00–0.20	509 (12.4)	252 (7.2)	257 (43.8)	—	509 (28.9)	252 (21.6)	257 (43.8)	—
0.21–0.50	212 (5.2)	86 (2.4)	126 (21.5)	—	212 (12.0)	86 (7.3)	126 (21.5)	—
>0.50	86 (2.0)	44 (1.2)	42 (7.1)	—	86 (4.9)	44 (3.6)	42 (7.1)	—

NOS, not otherwise specified; ACT, adjuvant chemotherapy; API, Asian/Pacific Islander; rN, metastastic lymph nodes ratio.

**FIGURE 2:**
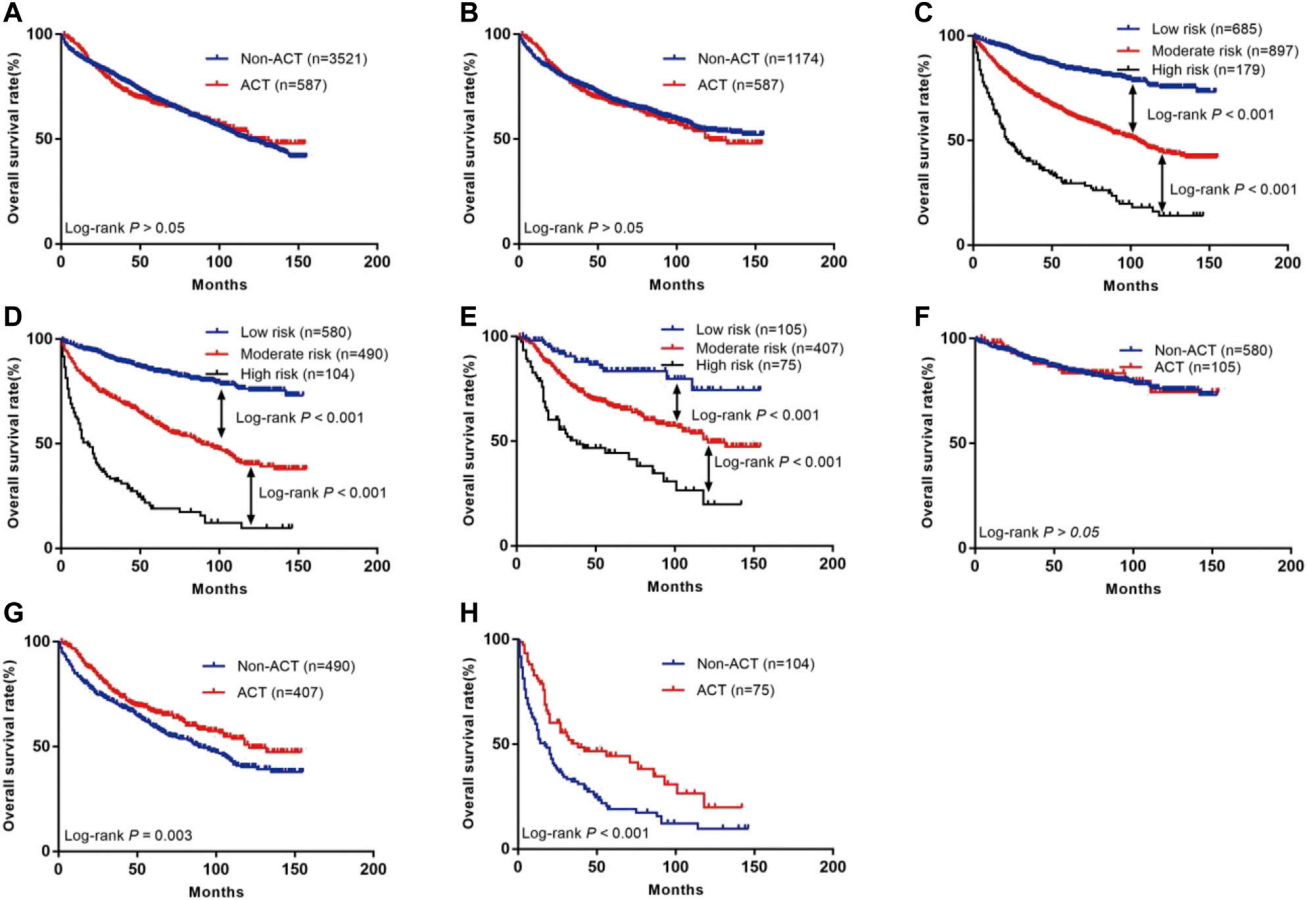
The Kaplan-Meier curves of OS for patients in our study. **(A)** All patients; **(B)** Patients after PSM; **(C)** OS in low, moderate and high risk subsets of non-ACT group and ACT group; **(D)** OS in different subsets of non-ACT group; **(E)** OS in different subsets of ACT group; **(F)** OS for patients with or without ACT in low risk group; **(G)** OS for patients with or without ACT in moderate risk group; **(H)** OS for patients with or without ACT in high risk group.

There was no difference in the prognosis between the non-ACT and ACT groups before PSM analysis (5-year survival rate of 69.2 vs. 66.9%, *p* > 0.05, Figure 2A). There was also no difference in the prognosis between the two groups after PSM analysis (5-year survival rate of 68.3 vs. 66.9%, *p* > 0.05, Figure 2B).

### Nomogram Construction

**FIGURE 3:**
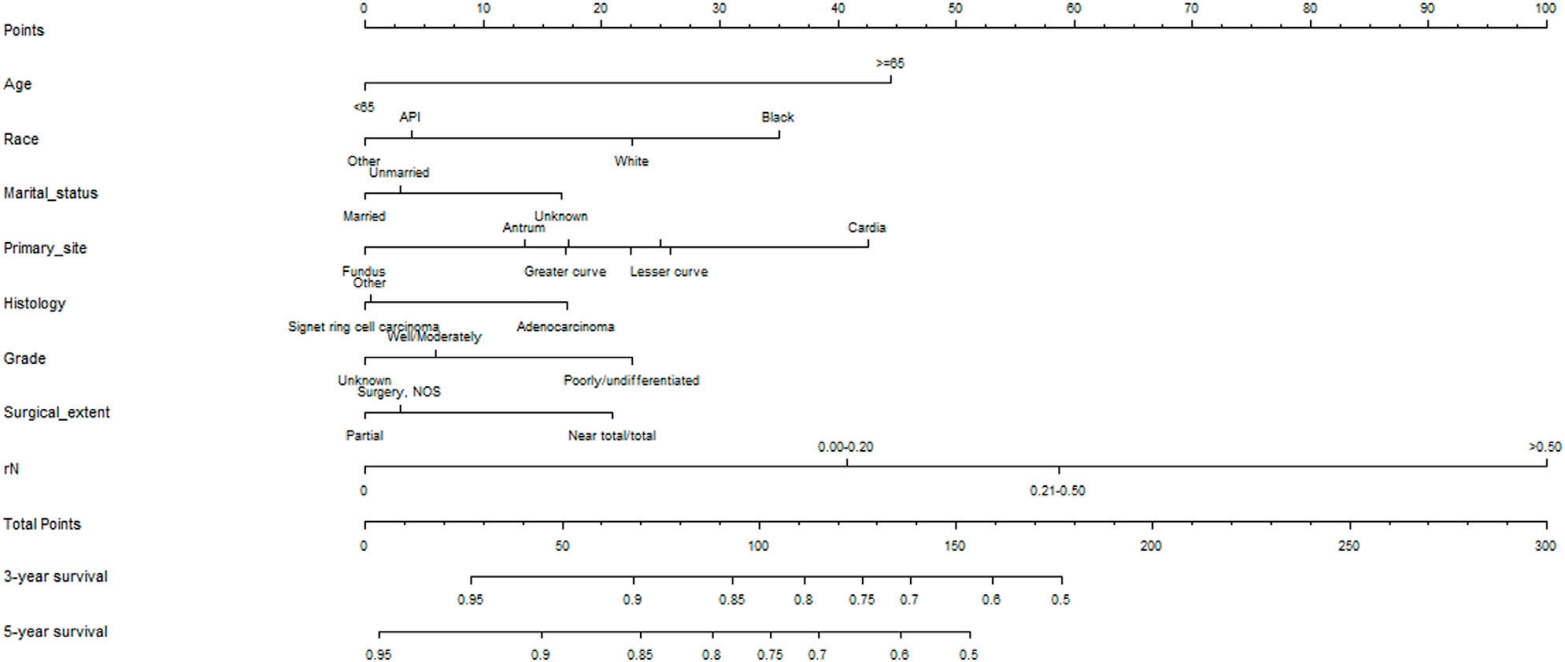
Prognostic nomograms for patients with surgery on early gastric cancer.

The data for patients who did not receive ACT after the operation were included in the COX proportional-hazards model (Table 2). Univariate analysis found that age, race, marital status, primary site, histology, grade, surgical extent, and rN were related to OS (*p* < 0.05). Further incorporating these variables into multivariate analysis found that age, race, marital status, primary site, histology, grade, surgical extent, and rN were independent prognostic factors (*p* < 0.05). A nomogram was constructed on this basis to predict the three- and 5-year OS rates for EGC (Figure 3).

**TABLE 2:** The univariate and multivariate analyses of factors associated with overall survival.

Variable	Univariate cox regression		Multivariate cox regression	
	HR (95% CI)	*p*-Value	HR (95% CI)	*p*-Value
Age				
<65	1	—	—	—
≥65	3.528 (2.997–4.154)	<0.001	2.122 (1.729–2.605)	<0.001
Sex				
Male	1	—	—	—
Female	0.911 (0.775–1.071)	0.911	—	—
Race				
White	1	—	—	—
Black	1.059 (0.858–1.308)	0.594	1.240 (0.989–1.555)	0.063
API	0.667 (0.557–0.797)	<0.001	0.735 (0.607–0.891)	0.002
Other	0.509 (0.252–1.027)	0.059	0.646 (0.317–1.317)	0.229
Marital status				
Married	1	—	—	—
Unmarried	1.217 (0.958–1.545)	0.107	1.063 (0.828–1.365)	0.632
Unknown	2.471 (2.008–3.042)	<0.001	1.363 (1.093–1.700)	0.006
Primary site				
Cardia	1	—	—	—
Fundus	0.509 (0.283–0.917)	0.024	0.472 (0.257–0.867)	0.015
Body	0.715 (0.544–0.940)	0.016	0.695 (0.521–0.927)	0.013
Antrum	0.614 (0.494–0.762)	<0.000	0.605 (0.472–0.775)	<0.001
Pylorus	0.795 (0.481–1.314)	0.371	0.729 (0.433–1.227)	0.234
Lesser curve	0.588 (0.440–0.784)	<0.001	0.679 (0.497–0.927)	0.015
Greater curve	0.940 (0.661–1.338)	0.732	0.748 (0.511–1.094)	0.748
Overlapping/not otherwise specified	0.754 (0.574–0.989)	0.042	0.703 (0.525–0.941)	0.018
Histology				
Adenocarcinoma	1	—	—	—
Signet ring cell carcinoma	0.581 (0.459–0.735)	<0.001	0.655 (0.508–0.844)	0.001
Other	0.598 (0.431–0.828)	0.002	0.656 (0.470–0.917)	0.014
Grade				
Well/moderately	1	—	—	—
Poorly/undifferentiated	0.996 (0.847–1.173)	0.966	1.323 (1.113–1.573)	0.002
Unknown	0.639 (0.451–0.906)	0.012	1.236 (0.862–1.774)	0.250
Size (cm)				
≤2	1	—	—	—
≤3	1.451 (1.190–1.771)	<0.001	—	—
≤5	1.569 (1.281–1.922)	<0.001	—	—
>5	1.912 (1.457–2.509)	<0.001	—	—
Surgical extent				
Partial	1	—	—	—
Near total/total	1.473 (1.202–1.805)	<0.001	1.543 (1.242–1.916)	<0.001
Surgery, NOS	1.154 (0.721–1.848)	0.551	1.000 (0.611–1.636)	0.999
T stage				
T1a	1	—	—	—
T1b	1.232 (1.000–1.516)	0.050	—	—
T1 NOS	1.460 (0.985–2.166)	0.060	—	—
TNM stage				
I	1	—	—	—
II	4.689 (3.263–6.738)	<0.001	—	—
III	6.119 (3.447–10.860)	<0.001	—	—
rN				
0	1	—	—	—
0.00–0.20	3.257 (2.721–3.899)	<0.001	2.126 (1.715–2.635)	<0.001
0.21–0.50	4.401 (3.462–5.595)	<0.001	2.931 (2.226–3.858)	<0.001
>0.50	6.733 (4.962–9.136)	<0.001	4.693 (3.331–6.613)	<0.001

NOS, not otherwise specified; API, Asian/Pacific Islander; rN, metastastic lymph nodes ratio.

### Testing Predictive Model Effectiveness

**FIGURE 4:**
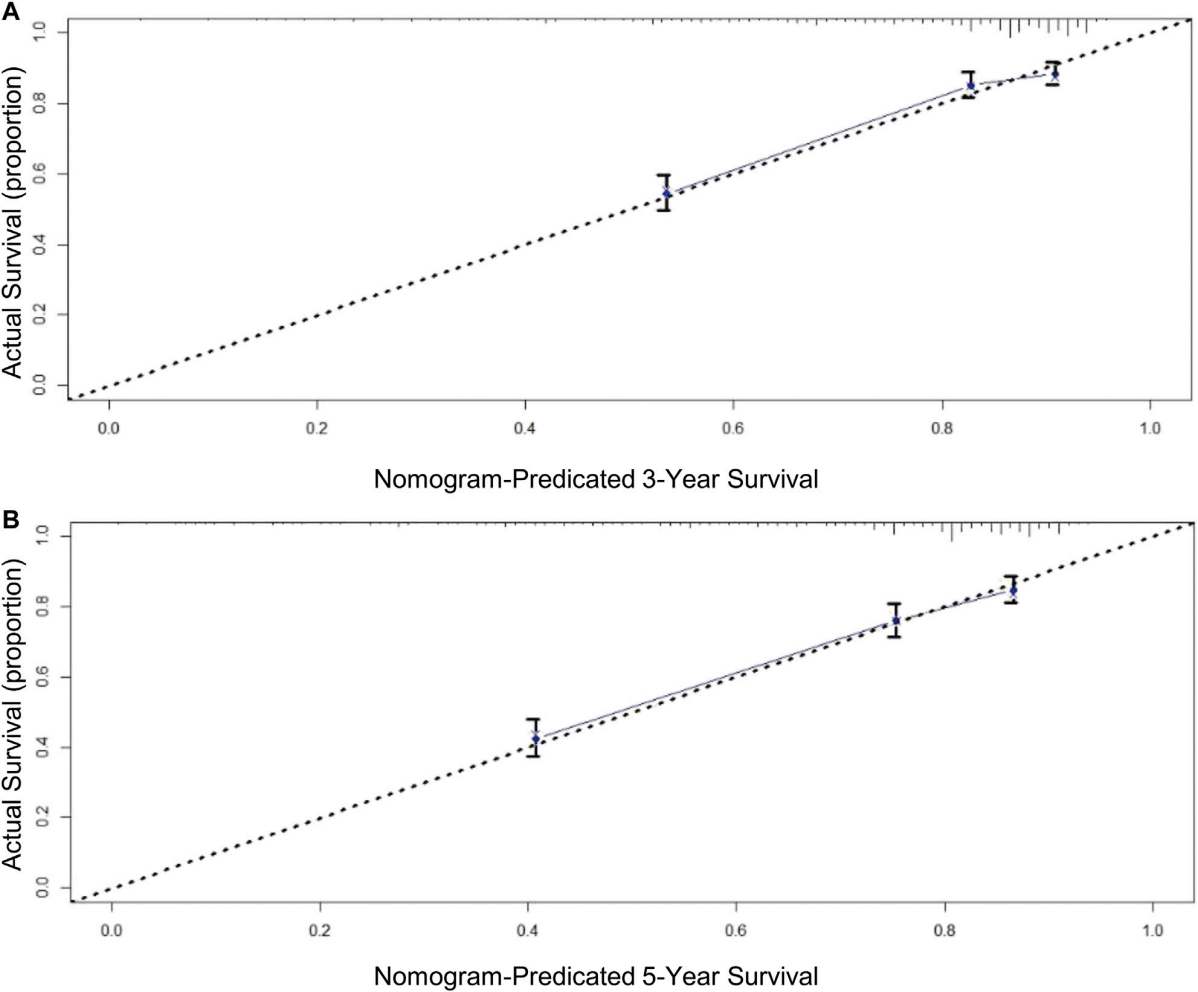
Calibration curves for OS prediction: **(A)** 3-year OS in derivation cohort; **(B)** 5-year OS in derivation cohort.

**FIGURE 5:**
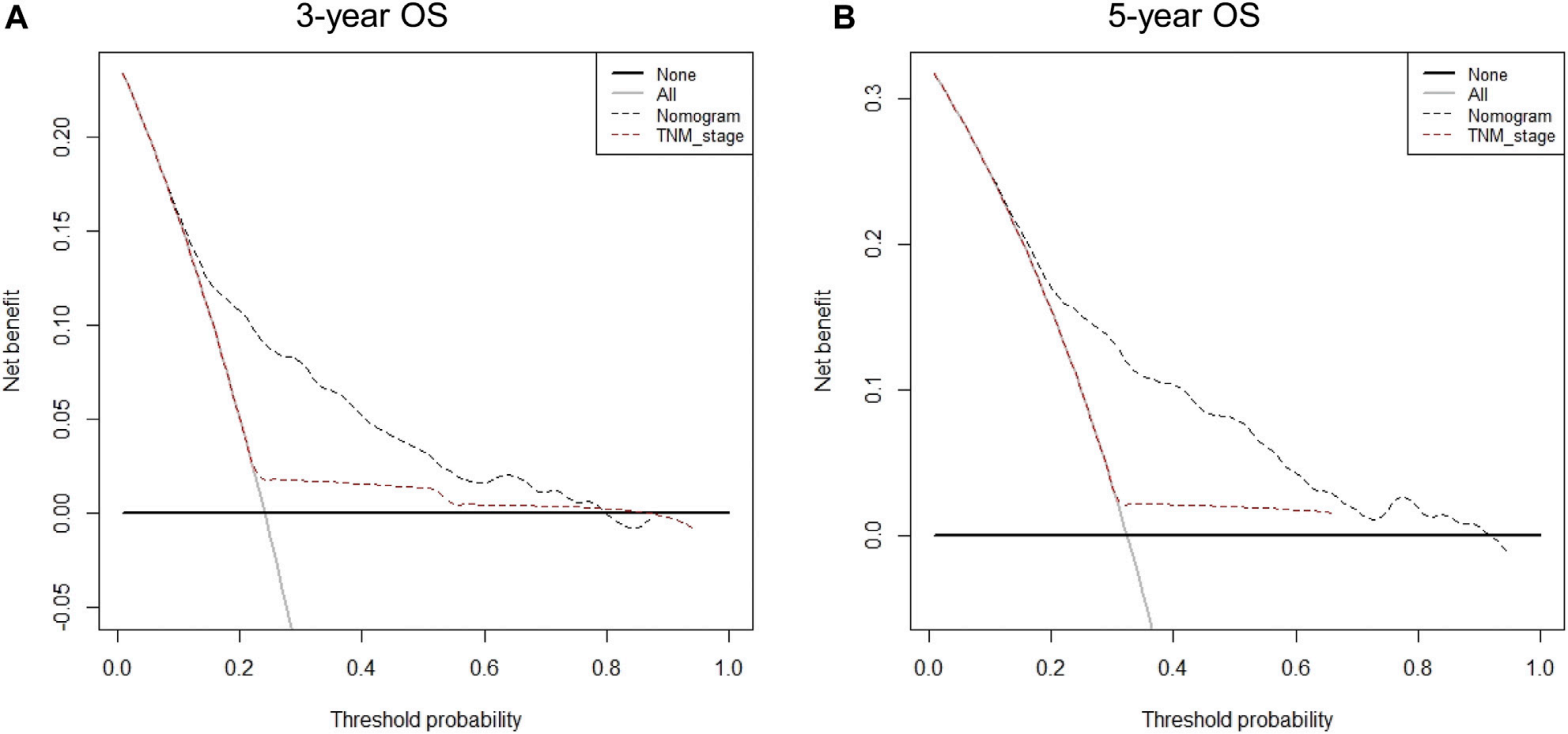
Decision curve analysis for different predictive models: **(A)** Nomogram were compared to the 8th AJCC TNM stage in terms of 3-year OS; **(B)** Nomogram were compared to the 8th AJCC TNM stage in terms of 5-year OS.

A total of eight variables were included in the non-ACT group to build a nomogram to predict the EGC prognosis. The nomogram C-index evaluating the prognosis was 0.730 (95% confidence interval (CI): 0.677–0.783), which was significantly higher than the 8th AJCC TNM stage C-index of 0.534 (95% CI: 0.507–0.561). Compared to the TNM staging system, a nomogram has a stronger ability to predict the OS and prognosis of EGC. The calibration curve for three- and 5-year OS shows that the predicted survival probability is in agreement with the actual survival probability (Figure 4). DCA revealed that the net benefit of the nomogram prognostic model for different decision thresholds was higher than the prediction line of the 8th AJCC TNM stage, which suggested a higher predictive power for three- and 5-year OS (Figure 5).

### Risk Stratification System

**FIGURE 6:**
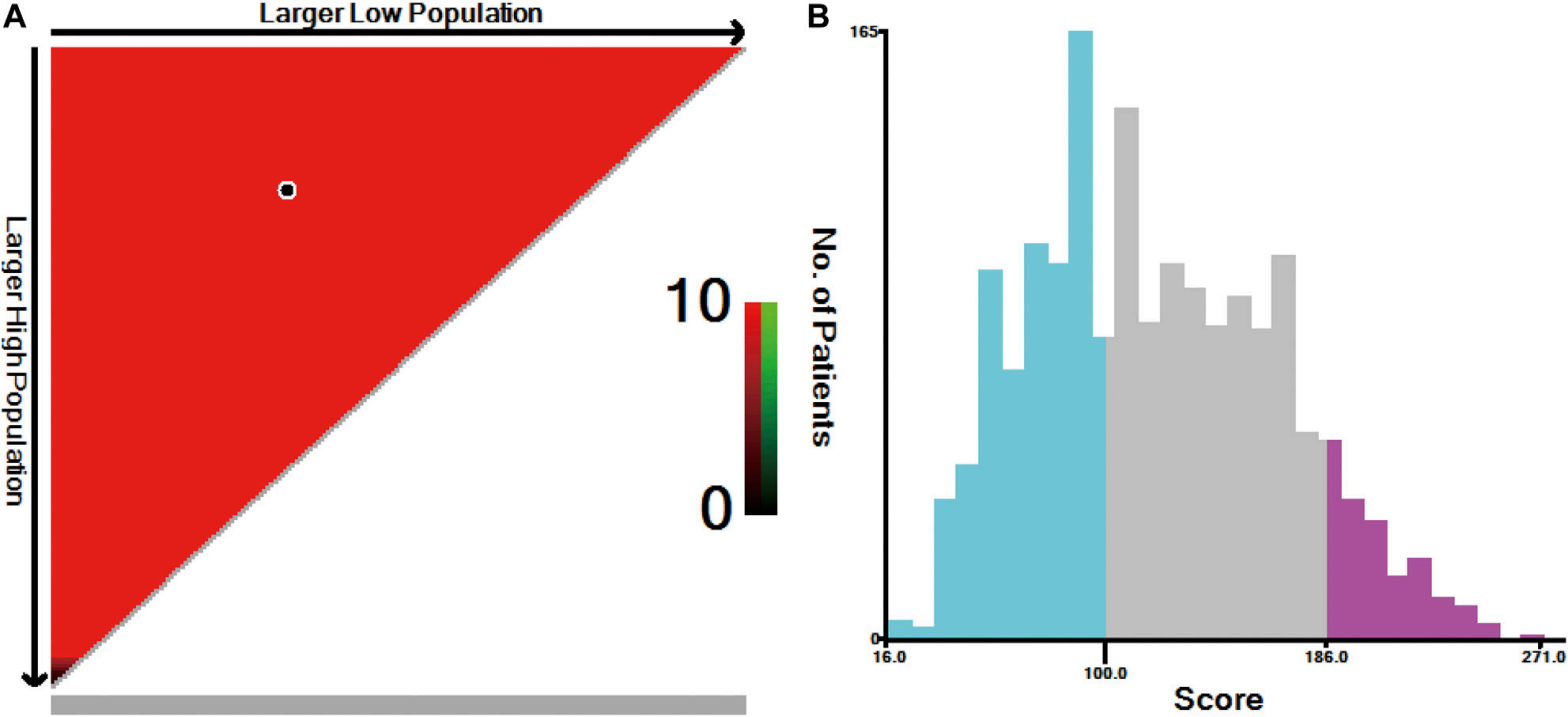
X-tile analysis for risk stratification: **(A)** The optimal cut-off value; **(B)** Numbers of patients in low, moderate and high risk subsets.

The individual risk score for all patients was calculated using the nomogram (Supplementary Table S1). X-tile software was used to divide all patients into three risk groups (Figure 6): the low- (score ≤100, *n* = 685), moderate- (score: 101–186, *n* = 897), and high-risk (score ≥187, *n* = 179) groups. The 5-year survival rates for the low-, moderate-, and high-risk groups were 84.3, 63.2, and 29.5%, respectively (*p* < 0.001, Figure 2C).

According to the Kaplan-Meier curve, the 5-year survival rates in the non-ACT low- (*n* = 580), moderate- (*n* = 490), and high-risk (*n* = 104) groups were 84.4, 59.6, and 19.0%, respectively (Figure 2D). This result was statistically significant (*p* < 0.001). According to the existing scoring system, the ACT group was divided into three subgroups of low- (*n* = 105), moderate- (*n* = 407), and high (*n* = 75) risk (Table 3). The 5-year survival rates for the low-, moderate-, and high-risk groups were 83.5, 66.9, and 44.3%, respectively (Figure 2E; *p* < 0.001).

**TABLE 3:** Risk stratification in non-ACT and ACT group.

Survival status	Non-ACT group				ACT group			
	Low risk [*n* (%)]	Moderate risk [*n* (%)]	High risk [*n* (%)]	*p* Value	Low risk [*n* (%)]	Moderate risk [*n* (%)]	High risk [*n* (%)]	*p* Value
Live	479 (82.6)	261 (53.3)	17 (16.3)	<0.001	89 (84.8)	266 (65.4)	31 (41.3)	<0.001
Death	101 (17.4)	229 (46.7)	87 (83.7)	—	16 (15.2)	141 (34.6)	44 (58.7)	—

ACT, adjuvant chemotherapy.

### Evaluating Adjuvant Chemotherapy Efficiency for Patients in Different Groups

The present study further compared whether low-, moderate-, and high-risk patients could benefit from ACT (Table 3). The results showed that patients in the low-risk group did not benefit from ACT (HR = 0.97; 95% CI: 0.58–1.64; *p* > 0.05; Figure 2F), while patients in the moderate- and high-risk groups did (HR = 0.73; 95% CI: 0.59–0.90; *p* = 0.003; Figure 2G; HR = 0.54; 95% CI: 0.38–0.99; *p* < 0.001; Figure 2H, respectively).

### Subgroup Analysis in the External Validation Group

External validation included 493 patients with EGC, including 323 in the non-ACT and 170 in the ACT groups (Supplementary Figure S2). The median patient survival time was 86 months (0–132), and the number of deaths was 146 (29.6%). The prognosis was better in the ACT groups than in the non-ACT groups (5-year survival rate of 90.5 vs. 88.8%, *p* < 0.05, Supplementary Figure S1A).

The patients were divided into three subgroups of low (*n* = 290), moderate (*n* = 127), and high (*n* = 76) risk according to the existing scoring system. The 5-year survival rates in the low-, moderate-, and high-risk groups were 94.0, 84.4, and 80.1%, respectively (Supplementary Figure S1B). The 5-year survival rates in the non-ACT low- (*n* = 203), moderate- (*n* = 80), and high-risk (*n* = 40) groups were 94.9, 82.6, and 69.9%, respectively (Supplementary Figure S1C). The 5-year survival rates in the ACT low- (*n* = 87), moderate- (*n* = 47), and high-risk (*n* = 36) groups were 91.8, 87.2, and 91.7%, respectively (Supplementary Figure S1D).

The results showed that patients in the low-risk group did not benefit from ACT (HR = 1.01; 95% CI: 0.60–1.68; *p* > 0.05; Supplementary Figure S1E), while patients in the moderate- and high-risk groups did (HR = 0.51; 95% CI: 0.28–0.93; *p* = 0.04; Supplementary Figure S1F; HR = 0.26; 95% CI: 0.13–0.55; *p* < 0.001; Supplementary Figure S1G, respectively).

## Discussion

Gastric cancer can be divided into EGC and advanced gastric cancer according to the degree of disease progression. EGC has a good prognosis, and its 5-year survival rate is significantly higher than that of advanced gastric cancer. Therefore, it is particularly important to improve EGC detection rate and provide effective treatment plans to reduce its mortality and improve the 5-year survival rate. Up until now, endoscopic resection and modified radical surgery have been accepted by many scholars. However, 2.7% of patients still experienced recurrence, and the recurrence rate in patients with lymph node metastasis reached ∼10% (Lai et al., 2009). Lymph node metastasis, invasion depth, and differentiation degree are all risk factors for EGC recurrence, while lymph node metastasis is the most important one. However, whether ACT can improve the prognosis of EGC patients with a high recurrence risk and poor prognosis, such as pT1N1, remains undecided (Shin et al., 2017; Kim et al., 2018). The economic burden and side effects of ACT are also factors that need to be considered. To achieve the maximum clinical benefit, patients with different outcomes require different treatment strategies. Therefore, the present study established a nomogram based on the SEER database and analyzed whether patients with different risk factors can benefit from ACT, hoping to provide a more accurate decision basis for the treatment of EGC patients.

Previous studies have shown that the factors affecting the prognosis of EGC include low degree of differentiation, lymph node metastasis, and advanced age (Cheong et al., 2006; Du et al., 2012). The risk factors were re-evaluated in the present study. Age, race, marital status, primary site, histology, grade, surgical extent, and rN were determined to be independent prognostic factors. Elderly patients have more complicated diseases, decreased organ function, greater surgical risk, more postoperative complications, and poor long-term prognosis. The present study found that prognosis for individuals aged ≥65 years was poor (HR = 2.122; 95% CI: 1.729–2.605; *p* < 0.001), which is consistent with previous research results (Jang et al., 2013). It was also found that the prognosis of cardiac EGC was poor compared to other sites. Patients with proximal gastric cancer had a high proportion of individuals aged ≥65 years, the tumor size was large, while the lymph node metastasis rate was high, all of which explain the poor prognosis (Petrelli et al., 2017; Yu et al., 2018). The Asian/Pacific Islander (API) prognosis was better than that of whites (HR = 0.735; 95% CI: 0.607–0.891; *p* = 0.002), which may be related to its lower incidence (Islami et al., 2019). Kunz et al. (Kunz et al., 2012) have also found that API had a better prognosis than that of whites (HR = 0.766; 95% CI: 0.727–0.806; *p* < 0.05), which was consistent with the present study. In cancer, unmarried status was also associated with poor prognosis, which may be related to the psychological and economic factors, as well as unwillingness to receive treatment (Li and Hu, 2019).

In the past, it was generally believed that signet ring cell carcinoma has a high degree of malignancy and a poor prognosis. However, continuous study of this type of disease found that signet ring cell carcinoma prognosis at different stages is significantly different. The advanced signet ring cell carcinoma prognosis is very poor, while early-stage prognosis is better than that of adenocarcinoma (Chon et al., 2017). Kim et al. (2017) believed that gastric signet ring cell carcinoma of pT1a is not associated with lymph node metastasis regardless of tumor size, while adenocarcinoma is an independent risk factor for lymph node metastasis. The present study also showed that signet ring cell carcinoma has a better prognosis than adenocarcinoma (HR = 0.655; 95% CI: 0.508–0.844; *p* = 0.001).

Undifferentiated EGC is highly malignant, aggressive, and prone to early metastasis. Feng et al. (Feng et al., 2016) have studied 976 EGC patients and found that the incidence of lymph node metastasis was 6.6% in differentiated and 20.5% in undifferentiated ECG patients. The present study also found that the prognosis of undifferentiated adenocarcinoma is worse than that of differentiated adenocarcinoma (HR = 1.323; 95% CI: 1.113–1.573; *p* = 0.002). In addition, patients with undifferentiated adenocarcinoma could not receive minimally invasive endoscopic treatment. The present study also found that expanded surgery results in poor prognosis (HR = 1.543; 95% CI: 1.242–1.916; *p* < 0.001). The larger the surgical extent, the greater the probability of patients undergoing unnecessary or excessive lymph node dissection and the greater the postoperative trauma and complications, which are also the reasons for poor prognosis in such patients.

Lymph node metastasis is considered to be one of the important factors affecting the prognosis of patients with gastric cancer. Accurate and reasonable lymph node staging is of great significance for determining the course of disease, evaluating the prognosis, and formulating a reasonable treatment plan. While inheriting and developing the classical lymph node staging system for gastric cancer, major research centers are also working on new methods of lymph node staging related to the prognosis of gastric cancer, such as using rN, which is defined as the number of positive lymph nodes/total number of lymph nodes dissected. A prognostic analysis of 1,075 gastric cancer patients performed by Zhou et al. (2013) indicated that no matter how many lymph nodes were examined, rN staging provided a better prediction of patient prognosis than N staging and suggested that rN staging should replace N staging to predict lymph node status. A study of 9,357 gastric cancer patients from the SEER database showed that in the vast majority of Western gastric cancer patients undergoing localized lymph node dissection, rN can effectively predict patient prognosis and can be divided into four stages: rN0 (0), rN1 (∼1–20%), rN2 (∼21–50%), and rN3 (∼51–100%) (Kutlu et al., 2015). This staging method was also used in the present study and found that the prognosis of rN1, rN2, and rN3 patients was worse than that of rN0 patients (HR = 2.126; 95% CI: 1.715–2.635; *p* < 0.001, HR = 2.931; 95% CI: 2.226–3.858; *p* < 0.001, HR = 4.693; 95% CI: 3.331–6.613; *p* < 0.001, respectively). Cheong et al. (Cheong et al., 2006) have found that the 5-year survival rates in EGC patients were 94.0 and 72.6% for rN < 0.07 and rN > 0.07, respectively. This is consistent with the present results. At the same time, the nomogram based on these prognostic factors has good discrimination and repeatability characteristics. The nomogram C-index was 0.730 (95% CI: 0.677–0.783), which was significantly higher than the C-index for the 8th AJCC TNM staging of 0.534 (95% CI: 0.507–0.561), indicating that the present study nomogram had a stronger predictive ability for EGC prognosis than the traditional TNM staging system. The DCA curve suggested better estimation for decision making.

It is controversial whether ACT should be performed after surgery in EGC. The present study showed that ACT did not have survival benefits in the whole patient before or after PSM analysis. This is mainly due to the fact that ACT is often used in clinical patients with poor prognostic factors, who also account for a small proportion of the total cohort. In this study, X-tile software was used to select two cutoff values (100 and 186) according to the patient’s risk score and divided the patients into low-, moderate-, and high-risk groups. In all of the patient group, including non-ACT and ACT groups, there was a significant difference in patient survival among low-, moderate-, and high-risk groups, indicating that risk stratification was reasonable and effective. While investigating who can really benefit from ACT, the 5-year survival rate in low-risk patients receiving ACT was found to be lower than that in patients who did not receive ACT (83.5 vs. 84.4%, *p* > 0.05). Therefore, ACT is not recommended for low-risk patients because its harmful effects outweigh its benefits. The 5-year survival rate in moderate-risk patients who received ACT was higher than that in patients who did not (66.9 vs. 59.6%, *p* < 0.01), indicating that moderate-risk patients can benefit from ACT. Patients in the high-risk group receiving ACT had a higher 5-year survival rate than those who did not receive ACT (44.3 vs.19.0%, *p* < 0.001), indicating that high-risk patients can benefit from ACT. The external validation also verified that high-risk patients can benefit from ACT. Therefore, ACT is suggested for moderate- and high-risk EGC patients after the operation.

The present research has several limitations. 1) This was a retrospective study, such as signet ring cell carcinoma or undifferentiated EGC has repeated parts, which may lead to bias. 2) The SEER database fails to provide specific ACT regimens, efficacy, and course of treatment, which affect the assessment of ACT efficacy. 3) Some EGC patients were not included in this study due to lack of data, leading to selection bias. 4) Progression-free survival was not evaluated in the present study, and other confounding factors may be involved in the overall survival, resulting in poor tumor survival assessment. 5) A nomogram that is applicable for the SEER and our center’s databases, as well as more studies from other centers, are needed. At present, no survival and prognosis model for EGC incorporates the above clinicopathological factors. Most importantly, this model was used to carry out risk stratification analysis for patients, which is of great significance for guiding clinical ACT. Since there has been no conclusion on whether EGC patients should receive ACT after surgery and the rate of EGC patients receiving ACT is generally low, it is difficult to carry out large RCT studies, which also highlights the importance of the present study.

## Conclusion

The nomogram in the present study can effectively predict EGC patient prognosis. Patients at moderate and high risk are recommended to receive ACT based on risk stratification analysis, while patients at low risk are not.

## Data Availability

The original contributions presented in the study are included in the article/Supplementary Material, further inquiries can be directed to the corresponding author.

## Supplementary Material

Supplementary Figure 1 The Kaplan-Meier curves of OS for patients in external validation: **(A)** All patients; **(B)** OS in low, moderate and high risk subsets of non-ACT group and ACT group; **(C)** OS in different subsets of non-ACT group; **(D)** OS in different subsets of ACT group; **(E)** OS for patients with or without ACT in low risk group; **(F)** OS for patients with or without ACT in moderate risk group; **(G)** OS for patients with or without ACT in high risk group.
