# Supplementary material for: Prognostic Nomogram for Early Gastric Cancer After Surgery to Assist Decision-Making for Treatment With Adjuvant Chemotherapy
Source: Front Pharmacol. 2022 Apr 8;13:845313. doi: 10.3389/fphar.2022.845313 (PMC9024108; doi:10.3389/fphar.2022.845313)
Supplement: Supplementary file 1 [file Table1.DOCX]

Table S1 Point assignment of each component and prognostic score for early gastric cancer

| Group | Score | Estimated 3-y OS (%) | Estimated 5-y OS (%) |
| --- | --- | --- | --- |
| Age |  |  |  |
| <65 | 0 |  |  |
| ≥65 | 44 |  |  |
| Race |  |  |  |
| White | 23 |  |  |
| Black | 35 |  |  |
| API | 4 |  |  |
| Other | 0 |  |  |
| Marital status |  |  |  |
| Married | 0 |  |  |
| Unmarried | 3 |  |  |
| Unknown | 17 |  |  |
| Primary site |  |  |  |
| Cardia | 43 |  |  |
| Fundus | 0 |  |  |
| Body | 17 |  |  |
| Antrum | 13 |  |  |
| Pylorus | 22 |  |  |
| Lesser curve | 26 |  |  |
| Greater curve | 17 |  |  |
| Overlapping/not otherwise specified | 25 |  |  |
| Histology |  |  |  |
| Adenocarcinoma | 17 |  |  |
| Signet ring cell carcinoma | 0 |  |  |
| Other | 0 |  |  |
| Grade |  |  |  |
| Well/moderately | 6 |  |  |
| Poorly/undifferentiated | 23 |  |  |
| Unknown | 0 |  |  |
| Surgical extent |  |  |  |
| Partial | 0 |  |  |
| Near total/total | 21 |  |  |
| Surgery, NOS | 3 |  |  |
| rN |  |  |  |
| 0 | 0 |  |  |
| 0.00-0.20 | 41 |  |  |
| 0.21-0.50 | 59 |  |  |
| >0.50 | 100 |  |  |
| Total score |  |  |  |
|  | 27 | 95 |  |
|  | 68 | 90 |  |
|  | 93 | 85 |  |
|  | 111 | 80 |  |
|  | 126 | 75 |  |
|  | 138 | 70 |  |
|  | 159 | 60 |  |
|  | 177 | 50 |  |
|  | 3 |  | 95 |
|  | 45 |  | 90 |
|  | 70 |  | 85 |
|  | 88 |  | 80 |
|  | 103 |  | 75 |
|  | 115 |  | 70 |
|  | 136 |  | 60 |
|  | 154 |  | 50 |

NOS, not otherwise specified; API, Asian/Pacific Islander; rN, metastastic lymph nodes ratio
