# Supplementary figures and images for: Prognostic Nomogram for Early Gastric Cancer After Surgery to Assist Decision-Making for Treatment With Adjuvant Chemotherapy
Source: Front Pharmacol. 2022 Apr 8;13:845313. doi: 10.3389/fphar.2022.845313 (PMC9024108; doi:10.3389/fphar.2022.845313)

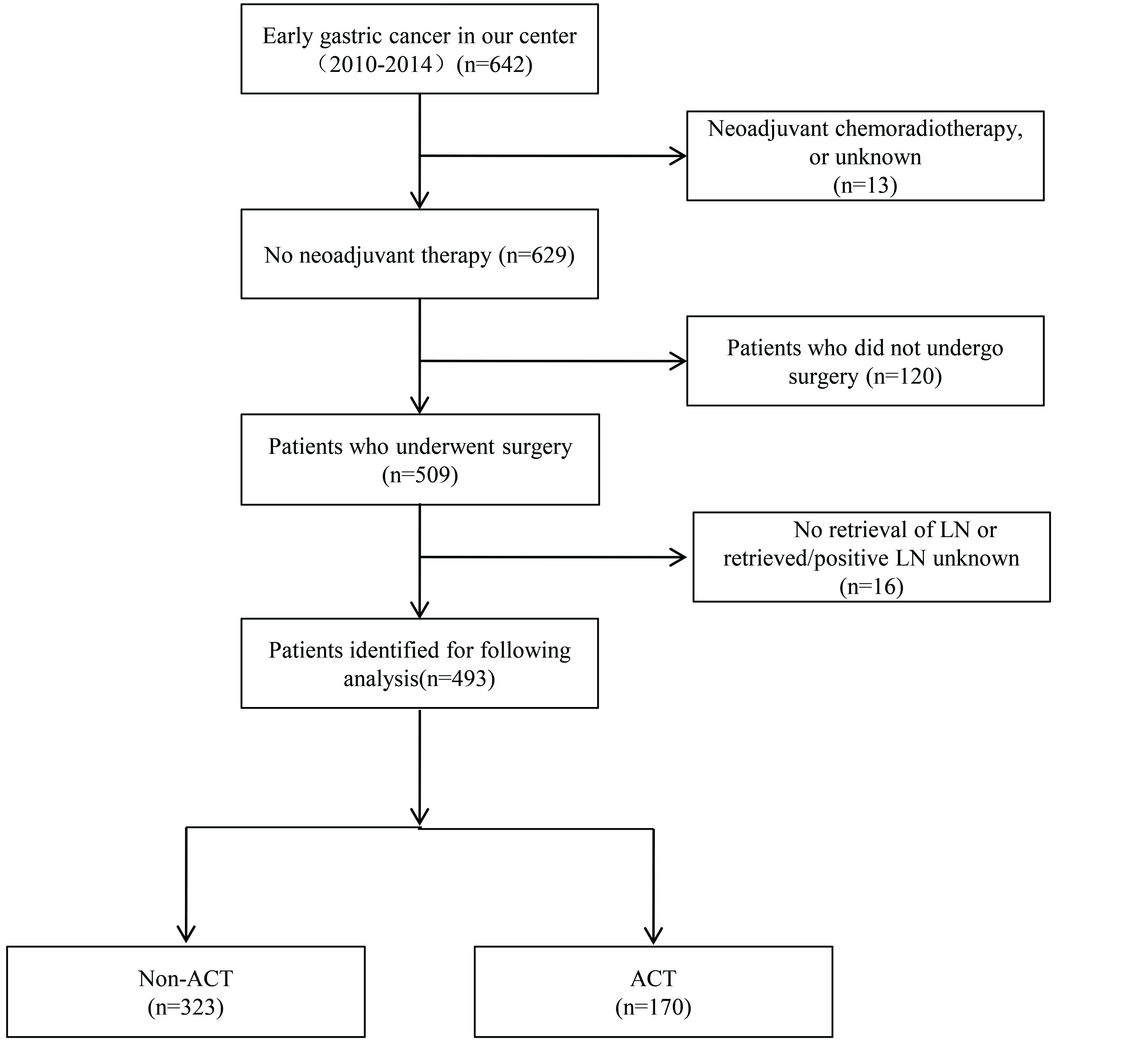

Supplement: Supplementary file 2 [file Image2.JPEG]

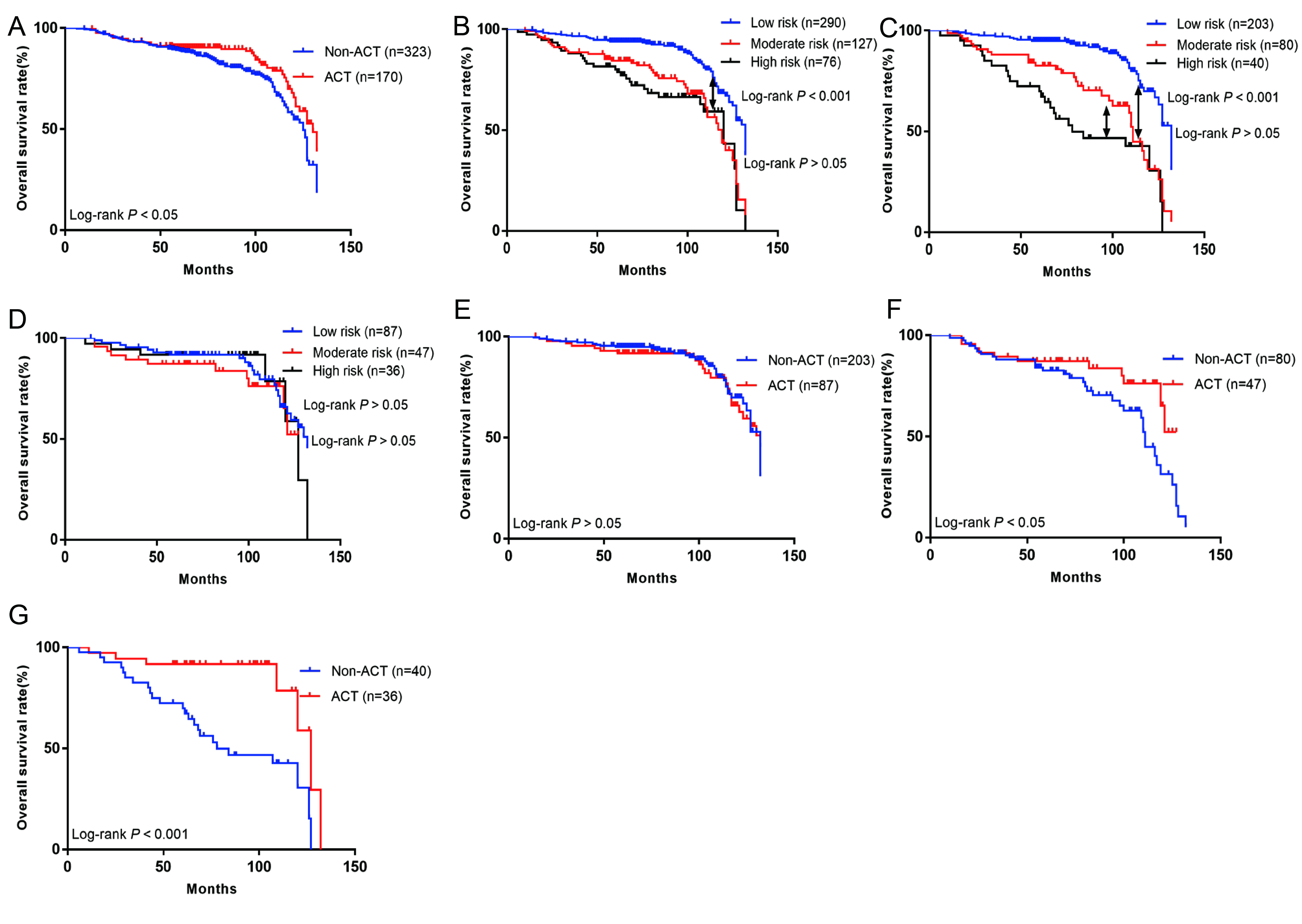

Supplement: Supplementary file 3 [file Image1.TIF]
